# Correction: Irisin directly stimulates osteoclastogenesis and bone resorption in vitro and in vivo

**DOI:** 10.7554/eLife.104341

**Published:** 2024-11-11

**Authors:** Eben G Estell, Phuong T Le, Yosta Vegting, Hyeonwoo Kim, Christiane Wrann, Mary L Bouxsein, Kenichi Nagano, Roland Baron, Bruce M Spiegelman, Clifford J Rosen

**Keywords:** Mouse

Estell EG, Le PT, Vegting Y, Kim H, Wrann C, Bouxsein ML, Nagano K, Baron R, Spiegelman BM, Rosen CJ. 2020. Irisin directly stimulates osteoclastogenesis and bone resorption in vitro and in vivo. *eLife* **9**:e58172. doi: 10.7554/eLife.58172.

Published 11 August 2020

The authors were made aware via a comment on PubPeer of an unintentional error of duplication in Figure 1—figure supplement 1. Upon reviewing the figure, we have confirmed that in the left panel of Alkaline Phosphatase staining, the bottom right image in the irisin (ISN) group is the same sample as the top left control (CTL) image. Noting differences in lighting, we consulted the raw images and confirmed that this unintentional duplication occurred when the control sample well was mistakenly imaged twice from different orientations and then included as the third irisin sample. The correct image of this irisin sample has been included in the updated figure below, and the raw image files provided to the editor. No changes to the text or legend associated with this figure were necessary, and the authors note that the interpretations and statements made in the paper both regarding this figure and in general remain unchanged.

The corrected Figure 1—figure supplement 1 is shown here:

**Figure 1:**
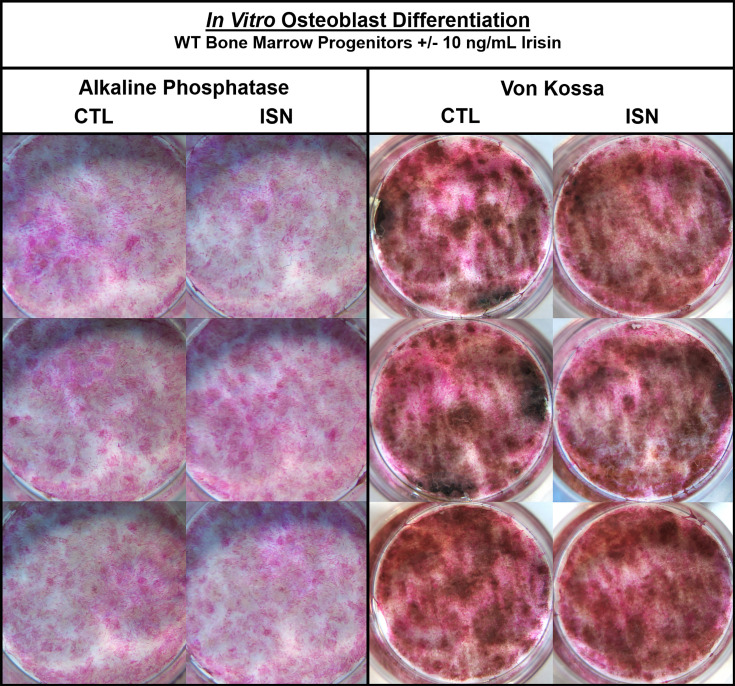


The originally published Figure 1—figure supplement 1 is shown for reference:

**Figure 2:**
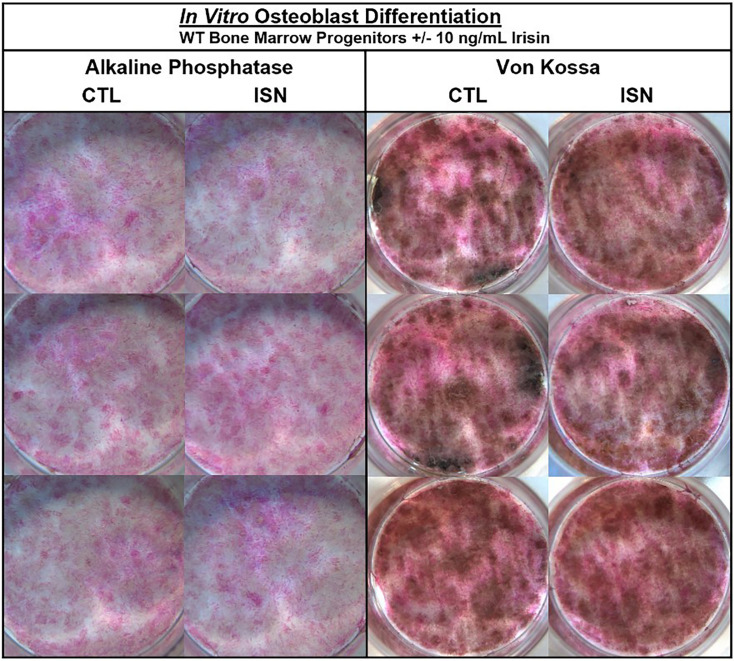


The authors would like to further update the Data availability statement to reflect the updated status of RNAseq data made available on the Dryad repository.

Corrected text:

Data availability statement: All data generated or analysed during this study are included in the manuscript figures and Source data 1, with the RNAseq dataset uploaded to the Dryad Digital Repository under “Eben Estell, Phuong T Le, Yosta Vegting, Hyeonwoo Kim, Christiane Wrann, Mary L Bouxsein, Kenichi Nagano, Roland Baron, Bruce M Spiegelman, Clifford Rosen (2020). Irisin directly stimulates osteoclastogenesis and bone resorption in vitro and in vivo: RNAseq dataset [Dataset]. Dryad Digital Repository, doi: 10.5061/dryad.hqbzkh1dr”.

Original text:

Data availability statement: All data generated or analysed during this study are included in the manuscript figures and Source data 1.

To further support reader accessibility to the comments made in PubPeer, the authors would like to address specific details and suggestions for correction below:

1. The primer sequence list has been uploaded as Supplementary file 1, with the following legend text:

Supplementary file 1. Primer Sequence List. Primer sequences used for quantitative RT-PCR on RNA isolated from irisin treated and control primary osteoclast cultures.

2. MCK-FNDC5 mouse line methodologies. The following correction has been made to the Methods section to provide further details on the generation and validation of the MCK-FNDC5 mouse line.

Corrected text:

Transgenic mice with muscle-specific forced expression of *Fndc5* were generated on a C57BL/6 J background utilizing a muscle creatine kinase (MCK) promoter as previously described for PGC1-a overexpression^17^, targeting the coding sequence of the mouse *Fndc5* gene. Transgenic mice were backcrossed to the C57BL/6 J background prior to experiments, and non-transgenic littermate controls were utilized for skeletal phenotyping.

Original text:

Transgenic C57BL/6 J mice with forced expression of *Fndc5* were generated and generously gifted by Dr. Eric Olson of UT Southwestern. The *Mck* promoter was utilized as previously demonstrated to induce skeletal muscle-specific forced expression of *Fndc5*.

3. In the summary of the peer-review file of this paper provided by the journal, Fndc5 is incorrectly described as an upstream regulator of irisin.

Corrected text:

Using a combination of in vitro, RNAseq, and in vivo studies (transgenic mouse model over-expressing Fndc5, the precursor protein of irisin), the authors provide strong evidence that, in addition to its known effects on osteoblasts and osteocytes, irisin directly regulates osteoclasts.

Original text:

Using a combination of in vitro, RNAseq, and in vivo studies (transgenic mouse model over-expressing Fndc5, an upstream regulator of Irisin in response to exercise), the authors provide strong evidence that, in addition to its known effects on osteoblasts and osteocytes, irisin directly regulates osteoclasts.

4. An unclear reference of irisin doses measured in prior human studies has been addressed with more details of the experimental design approach in the Discussion.

Corrected text:

Our previous work employed mass spectrometry to demonstrate that irisin concentrations in humans are approximately 2–4 ng/mL (Jedrychowski et al., 2015). As direct quantification of irisin protein levels via ELISA remains a challenge for the field, we used this range measured via mass spectrometry to guide our selection of irisin doses. While we primarily employed a concentration of 10 ng/mL to represent a robust but physiologically relevant in vitro stimulus, we also demonstrated that even low concentrations down to 2 ng/mL significantly increase osteoclast number.

Original text:

Our previous work demonstrated that irisin concentrations in humans are approximately 2–4 ng/mL but increase to 10 ng/mL or more with exercise, a dose range we employed for the in vitro studies (Jedrychowski et al., 2015).

The article has been corrected accordingly.

