# Correction: Irisin directly stimulates osteoclastogenesis and bone resorption in vitro and in vivo

**DOI:** 10.7554/eLife.112997

**Published:** 2026-09-10

**Authors:** Eben G Estell, Phuong T Le, Yosta Vegting, Hyeonwoo Kim, Christiane Wrann, Mary L Bouxsein, Kenichi Nagano, Roland Baron, Bruce M Spiegelman, Clifford J Rosen

**Keywords:** Mouse

Estell EG, Le PT, Vegting Y, Kim H, Wrann C, Bouxsein ML, Nagano K, Baron R, Spiegelman BM, Rosen CJ. 2020. Irisin directly stimulates osteoclastogenesis and bone resorption in vitro and in vivo. *eLife* **9**:e58172. doi: 10.7554/eLife.58172.

Published 11 August 2020

Statement on Impact to Conclusions and Reproducibility.

The following outlines and corrects a specific error found in one supplemental image, and addresses additional methodological questions from the scientific community. The authors would like to assert that the original error and its subsequent correction have no impact on the central findings of the paper, where in vitro and in vivo data demonstrate that irisin stimulates osteoclast differentiation. The supplemental experiment was conducted in osteoblasts. The absence of an effect is not presented as evidence to rule out the action of irisin on this cell type, but rather to inform future experiments to determine under what conditions irisin may signal to all bone cell types. The authors have addressed additional comments to ensure adequate detail is provided to support the reproducibility of this work moving forward.

1. Correction to Figure 1—figure supplement 1

The authors were made aware via comments on PubPeer of an unintentional error of duplication made during the correction of a similar error in the originally published Figure 1—figure supplement 1. This first correction made in November 2024 presented a revised figure and addressed additional comments on PubPeer. Please refer to the first Correction Notice (https://doi.org/10.7554/eLife.104341) for these initial corrections, and the full comments and author responses on PubPeer (https://pubpeer.com/publications/604C183AA357F5C9C89B96BC2EB211).

In the originally published figure the bottom irisin (ISN) panel for Alkaline Phosphatase was a duplicate of the top control (CTL) panel, as a result of the same well being imaged twice in different orientations. In the initial correction, this bottom ISN panel was mistakenly replaced with an image that was similarly a duplicate of the bottom CTL panel, via the same repeat imaging issue. In this second correction, we identify this error in the original and corrected individual well images, and present a full-plate image of the experiment to address the issue.

As detailed in the original correction, we determined to the best of our ability that this error was made during imaging where the same well was inadvertently imaged twice in different orientations, leading to subsequent image files being mis-labelled. When working on the correction, we believed this error was limited to a single well, and endeavoured to identify the correct ‘ISN-3’ image based on identifying features in the raw images. However, upon more closely reviewing this set of images we have determined multiple wells were affected, leading to the repeated error.

For simplicity in the original supplement, we included only the 10 ng/mL irisin dose from this experiment as the standard dose used across other experiments. The full experiment included 0.2 and 50 ng/mL irisin doses that also showed no qualitative effect on osteoblast differentiation. This well plate was stained for alkaline phosphatase and then von Kossa, with individual well images taken after each stain. Upon closer comparison of all raw images via semi-transparent overlapping (see PubPeer response), we have determined that during imaging after alkaline phosphatase staining, the entire row of 0.2 ng/mL irisin wells were mistakenly imaged twice in lieu of the control column, as well as mis-labelling between a pair of 0.2 and 10 ng/mL irisin wells. This led to the original figure showing mis-labelled ‘CTL’ wells in addition to the originally noted error of the ‘ISN-3’ image being a duplicate of one of these wells, an error which then occurred again when the correction was made. While the figure in its current form cannot be corrected as it is lacking properly identified control images for the alkaline phosphatase stage of the experiment, we have a final image of the entire well plate after both the alkaline phosphatase and von Kossa staining were done. As this image provides a complete, uncropped view of all experimental groups with alkaline phosphatase stain visible in un-mineralized areas and von Kossa staining clearly visible, we believe it still provides adequate qualitative evidence that these irisin treatments did not affect osteoblast function.

The full-plate image has been included as the corrected figure below, and the raw image files provided as Figure 1—figure supplement 1—source data 1.

The corrected Figure 1—figure supplement 1 is shown here:

**Figure 1:**
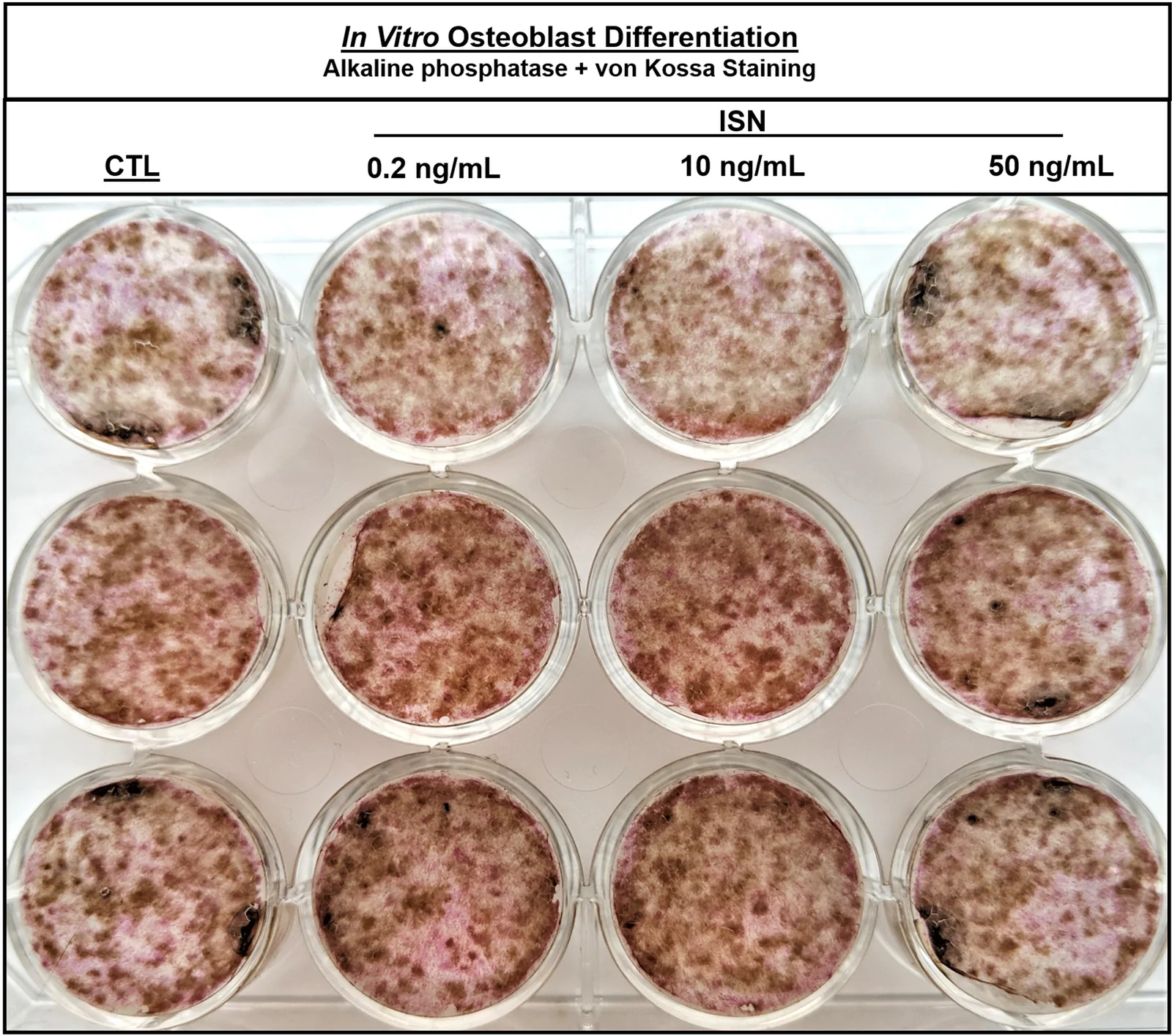


The previously corrected Figure 1—figure supplement 1 is shown here for reference:

**Figure 2:**
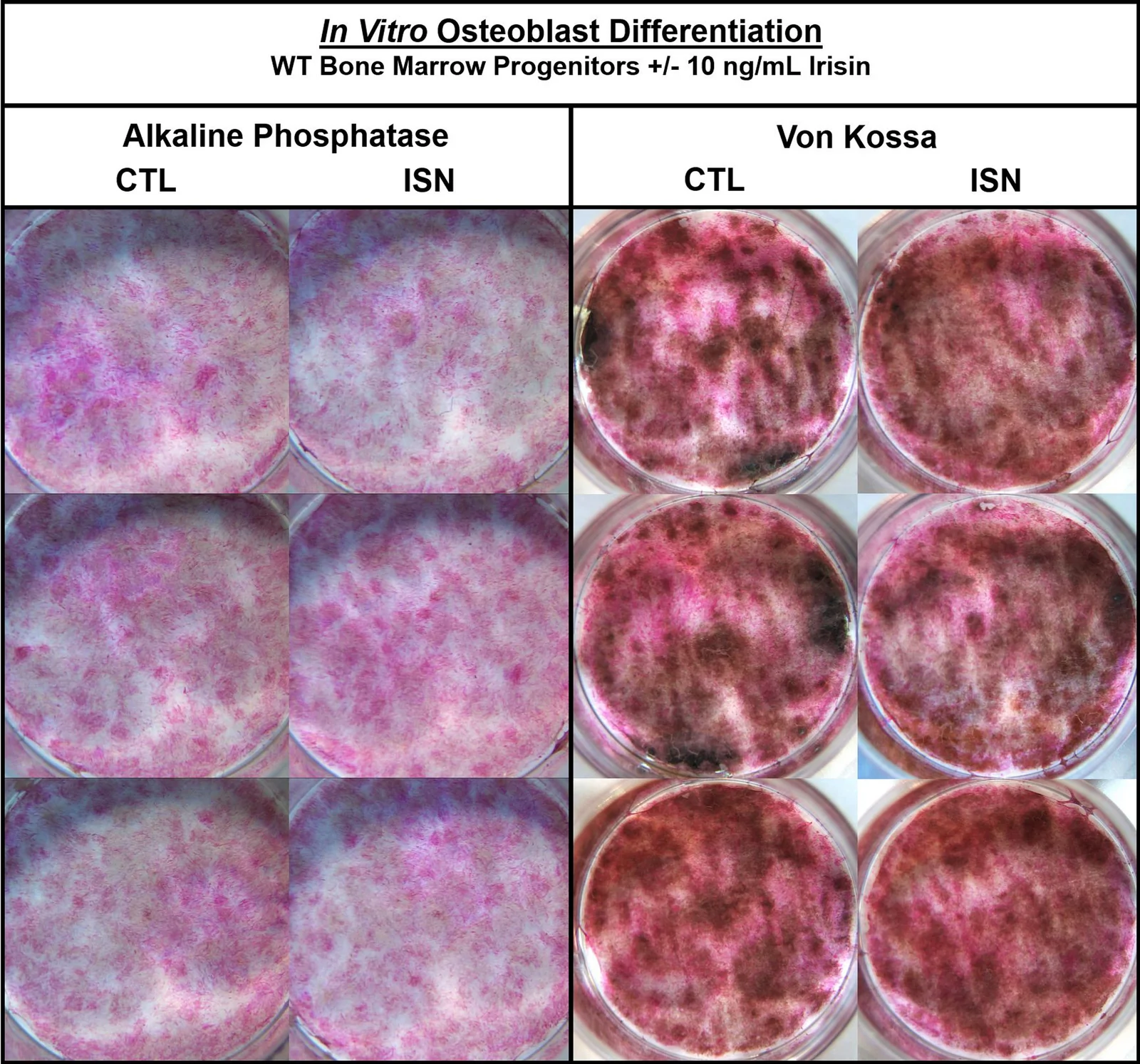


Minor changes to the legend associated with this figure were made to reflect the additional groups and nature of the dual staining being shown.

Corrected Figure 1—figure supplement 1 legend:

Representative images of in vitro osteoblast cultures with no (CTL) or 0.2, 10, or 50 ng/mL irisin (ISN) during 18-day differentiation.

Dual-staining for Alkaline phosphatase (pink) and subsequent von Kossa (brown) shows no qualitative effect of irisin doses on mineralization.

Original Figure 1—figure supplement 1 legend:

Representative images of in vitro osteoblast cultures with (ISN) or without (CTL) 10 ng/mL irisin during 18-day differentiation.

Alkaline phosphatase and von Kossa staining show no qualitative effect of irisin on differentiation or mineralization, respectively.

Minor changes to the sentence in the Results and Discussion addressing this experiment have been made to reflect the updated figure. The authors note that interpretations and statements made in the paper both regarding this specific figure and its main findings remain unchanged and unaffected.

Corrected Text in Results and Discussion:

Additionally, we have found that continuous treatment of osteoblast cultures from wild type progenitors with 0.2, 10, or 50 ng/mL exogenous irisin had no observable effect on differentiation or mineralization (Figure 1—figure supplement 1).

Original Text in Results and Discussion:

Additionally, we have found that continuous exogenous treatment with 10 ng/mL irisin used to stimulate in vitro osteoclast differentiation from wild type progenitors had no observable effect on osteoblast differentiation or mineralization (Figure 1—figure supplement 1).

2. Addressing Additional Questions on PubPeer

In addition to the PubPeer comments previously addressed in the first Correction Notice (https://doi.org/10.7554/eLife.104341), the following correction has been made to the Discussion section to better address the limitations of the mouse model, and outline the ongoing work of its characterization:

Corrected text:

To confirm the capacity of irisin to impact osteoclast function in vivo, we employed a genetic strategy with chronically forced expression of *Fndc5* using the muscle-specific *Mck* promoter. Interestingly, we found that this promoter resulted in increased gene expression of *Fndc5* in the bone itself. While this still establishes a model where bone cells would be exposed to elevated levels of irisin, future work will utilize other Cre models to address the potential leakiness of the *McK* promoter and investigate muscle-released irisin specifically, as well as develop more accurate analysis techniques to quantify irisin protein levels resulting from increases in precursor expression. In addition to these limitations, in vivo models such as this cannot fully isolate the relative contribution of osteoclasts and resorption to the overall skeletal phenotype. They can however provide key insights into cell function within their native tissue, informing further in vitro work that together form a clearer picture of irisin’s net effect on bone remodeling.

Original text:

To confirm the capacity of irisin to impact osteoclast function in vivo, we employed a genetic strategy with chronically forced expression of *Fndc5* using the muscle-specific *Mck* promoter. In that mouse model, we demonstrated that high levels of irisin were expressed in the whole bone marrow at both ages, and this was associated with low bone mass. The relative contribution of osteoclastogenesis to the skeletal phenotype cannot be resolved in our genetic model.

