# Supplementary material for: Irisin directly stimulates osteoclastogenesis and bone resorption in vitro and in vivo
Source: eLife. 2020 Aug 11;9:e58172. doi: 10.7554/eLife.58172 (PMC7444909; doi:10.7554/eLife.58172)
Supplement: Source data 1. [file elife-58172-data1.docx]

**Irisin Directly Stimulates Osteoclastogenesis and Bone Resorption *In Vitro* and *In Vivo***

**Supplementary File: Figure Source Data Tables**

**Figure 1 – Source Data 1.** Descriptive statistics reported as mean ± standard deviation for quantitative data represented in Figure 1.

| **Figure** | **Group** | **Mean±SD** | **Metric** |
| --- | --- | --- | --- |
| **1b** | CTL | 126±7 | Osteoclast/Well |
|  | 2 ng/mL | 259±26 |  |
|  | 5 ng/mL | 232±22 |  |
|  | 10 ng/mL | 272±19 |  |
|  | 20 ng/mL | 169±40 |  |
| **1c** | CTL | 181±16 | Osteoclast/Well |
|  | 4hr | 210±23 |  |
|  | 24hr | 225±10 |  |
|  | 7d | 255±18 |  |
| **1d** | BL6-F CTL | 117±18 | Osteoclast/Well |
|  | BL6-F ISN | 167±25 |  |
|  | RAW CTL | 117±23 |  |
|  | RAW ISN | 162±52 |  |
| **1f** | ITGAV CTL | 1.0±0.2 | Relative mRNA |
|  | ITGAV ISN | 1.93±2.49 |  |
|  | ITGB5 CTL | 1.0±0.1 |  |
|  | ITGB5 ISN | 10.67±5.12 |  |
| **1g** | CTL/CTL | 159 ± 10 | Osteoclast/Well |
|  | CTL/ISN | 228 ± 56 |  |
|  | CTL/IgG | 143 ± 27 |  |
|  | ISN/IgG | 219 ± 47 |  |
|  | CTL/AVB5-AB | 125±7 |  |
|  | ISN/AVB5-AB | 152±29 |  |

**Figure 2 – Source Data 1.** Descriptive statistics reported as mean ± standard deviation for quantitative data represented in Figure 2.

| **Figure** | **Group** | **Mean±SD** | **Metric** |
| --- | --- | --- | --- |
| **2b** | CTL | 155±98 | Osteoclast/Slice |
|  | ISN | 415±113 |  |
|  | CTL | 3.2±1.4 | Resorption % Area |
|  | ISN | 0.83±0.15 |  |
|  | CTL | 0.008±0.003 | Resorption %/Cells |
|  | ISN | 0.008±0.004 |  |
| **2c** | CTL | 0.42±0.35 | Resorption % Area |
|  | ISN | 0.80±0.33 |  |
| **2d** | CTL | 4.78±1.2 | Relative Fluorescence Units (×10^3^) |
|  | 24 hr ISN | 10.42±1.5 |  |
|  | 72 hr ISN | 7.32±0.83 |  |

**Figure 3 – Source Data 1.** Significantly upregulated (right) and downregulated (left) genes with irisin treatment versus untreated controls identified by unbiased RNAseq analysis, with markers highlighted for upregulated resorption (orange) and clastokines (green), and downregulated macrophage (yellow) and lymphocyte (blue) differentiation. N = 3 samples/group, *P < .05, **P < .01, ***P < .001, ****P < .0001 vs. CTL.

| **Differentially Regulated Genes: Irisin versus Control** | | | |
| --- | --- | --- | --- |
|  | |  | |
| **Gene** | **Fold**  **Change** | **Gene** | **Fold**  **Change** |
| MST1R* | -1.28 | TIMP3**** | 1.53 |
| DAGLB* | -1.30 | CD4*** | 1.49 |
| CD9* | -1.30 | POSTN*** | 1.48 |
| FXYD2* | -1.32 | PF4*** | 1.48 |
| TTN* | -1.34 | SLC16A9** | 1.46 |
| H2-AB1* | -1.35 | HAVCR2** | 1.41 |
| OLFM1* | -1.36 | CLEC4N*** | 1.39 |
| ZFYVE28* | -1.36 | TBC1D4** | 1.38 |
| SLC5A3* | -1.36 | FBLN5* | 1.38 |
| SLC45A3** | -1.36 | NGP* | 1.37 |
| CD72** | -1.36 | MYH10** | 1.37 |
| AQP1* | -1.37 | IGFBP5* | 1.37 |
| QPCT* | -1.38 | TGFB2** | 1.37 |
| HPGD* | -1.39 | COL11A1* | 1.36 |
| SLC6A12**** | -1.43 | ADAMTS5* | 1.36 |
| SLAMF8** | -1.43 | **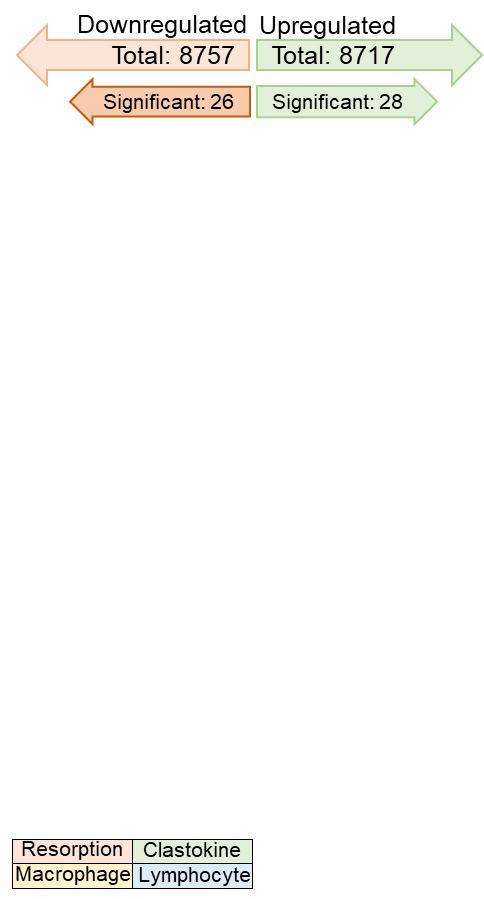**CTLA2B* | 1.36 |
| H2-AA** | -1.44 | OLFML2B* | 1.35 |
| EGR2** | -1.44 | MS4A4A** | 1.35 |
| GPX1**** | -1.45 | LOXL2** | 1.35 |
| ABCG1**** | -1.47 | CD33* | 1.35 |
| CD74**** | -1.47 | PDZRN3* | 1.34 |
| S100A4**** | -1.52 | ITGA11* | 1.32 |
| EEF1A2*** | -1.53 | COL5A2* | 1.30 |
| ITGAX**** | -1.53 | SPARC* | 1.29 |
| MAMDC2**** | -1.55 | CCDC80* | 1.28 |
| SLCO4A1**** | -1.68 | MS4A7* | 1.28 |
|  | | FCGR2B* | 1.27 |
|  |  | MRC1* | 1.27 |

**Figure 3 – Source Data 2.** Descriptive statistics reported as mean ± standard deviation for quantitative data represented in Figure 3. All data are in units of relative mRNA expression as normalized to *Hprt.*

| **Group** | **Mean±SD** | **Group** | **Mean±SD** |
| --- | --- | --- | --- |
| *Atp6vod2-CTL* | 1.02±0.19 | *Clcn7-CTL* | 1.15±0.61 |
| *Atp6vod2-ISN* | 2.85±1.48 | *Clcn7-ISN* | 1.43±1.37 |
| *Cfos-CTL* | 1.0±0.18 | *Csrc-CTL* | 1.18±0.86 |
| *Cfos-ISN* | 1.5±1.41 | *Csrc-ISN* | 4.2±2.07 |
| *Dcstamp-CTL* | 1.12±0.57 | *Ctsk-CTL* | 1.03 ± 0.27 |
| *Dcstamp-ISN* | 10.44±5.17 | *Ctsk-ISN* | 2.32 ± 2.05 |
| *Fam102a-CTL* | 1.1±0.62 | *Loxl2-CTL* | 1.0±0.16 |
| *Fam102a-ISN* | 1.95±0.61 | *Loxl2-ISN* | 12.4±8.41 |
| *Itgb3-CTL* | 1.72±0.92 | *Mmp2-CTL* | 1.02±0.28 |
| *Itgb3-ISN* | 11.34±11.11 | *Mmp2-ISN* | 3.27±2.82 |
| *Nfatc-CTL* | 1.0±0.14 | *Cthrc1-CTL* | 1.03±0.24 |
| *Nfatc-ISN* | 2.08±1.51 | *Cthrc1-ISN* | 1.57±0.90 |
| *Nrf2-CTL* | 1.03±0.27 | *Postn-CTL* | 1.17±0.61 |
| *Nrf2-ISN* | 2.9 ± 0.93 | *Postn-ISN* | 4.07±3.87 |
| *Rank-CTL* | 1.02±0.2 | *Sparc-CTL* | 1.05±0.28 |
| *Rank-ISN* | 4.37±2.93 | *Sparc-ISN* | 2.12±0.76 |
| *Rela-CTL* | 1.0±0.13 | *Tgfb2-CTL* | 1.07±0.40 |
| *Rela-ISN* | 1.57±0.82 | *Tgfb2-ISN* | 1.65±0.67 |
| *Rgs12-CTL* | 1.08±0.49 | *Wnt1-CTL* | 1.18±0.7 |
| *Rgs12-ISN* | 39.7±38.7 | *Wnt1-ISN* | 189.98±265.8 |
| *Acp5-CTL* | 1.15±0.76 | *Wnt5a-CTL* | 1.14±0.54 |
| *Acp5-ISN* | 11.3±5.88 | *Wnt5a-ISN* | 80.05±91.67 |
| *Adamts5-CTL* | 1.1±0.52 | *Wnt10a-CTL* | 1.03±0.25 |
| *Adamts5-ISN* | 127.7±139.1 | *Wnt10a-ISN* | 3.93±1.75 |

**Figure 4 – Source Data 1.** Descriptive statistics reported as mean ± standard deviation for quantitative data represented in Figure 4.

| **Figure** | **Group** | **Mean±SD** | **Metric** |
| --- | --- | --- | --- |
| **4a** | WT – 4.5 Month | 1.220±0.936 | Relative mRNA |
|  | TG – 4.5 Month | 1721.9±481.8 |  |
|  | WT – 13 Month | 0.820±0.811 |  |
|  | TG – 13 Month | 644.6±373.6 |  |
| **4b** | WT – 2 Month | 31.01±3.02 | BV/TV (%) |
|  | TG – 2 Month | 17.51±2.7 |  |
|  | WT – 4.5 Month | 24.1±2.38 |  |
|  | TG – 4.5 Month | 16.16±3.02 |  |
|  | WT – 13 Month | 7.68±2.67 |  |
|  | TG – 13 Month | 6.72±4.32 |  |
|  | WT – 2 Month | 0.164±0.006 | Ct.Th (mm) |
|  | TG – 2 Month | 0.136±0.01 |  |
|  | WT – 4.5 Month | 0.175±0.008 |  |
|  | TG – 4.5 Month | 0.182±0.012 |  |
|  | WT – 13 Month | 0.178±0.008 |  |
|  | TG – 13 Month | 0.169±0.007 |  |
| **4d** | WT | 304±35 | Osteoclast/Well |
|  | TG | 401±33 |  |

**Figure 4 – Source Data 2.** Quantitative data corresponding to representative images for µCT in Figure 4b. Descriptive statistics reported as mean ± standard deviation.

| **µCT**  **Bone**  **Architecture** | | **Two-way ANOVA**  **Significance Level** | | | **2 Months** | | | **4.5 Months** | | | **13 Months** | | |
| --- | --- | --- | --- | --- | --- | --- | --- | --- | --- | --- | --- | --- | --- |
|  |  |  |  |  | **WT** | **TG** | **Significance Level** | **WT** | **TG** | **Significance Level** | **WT** | **TG** | **Significance Level** |
|  |  | **Interaction** | **Age** | **Genotype** |  |  |  |  |  |  |  |  |  |
|  |  |  |  |  | **Mean±SD** | **Mean±SD** |  | **Mean±SD** | **Mean±SD** |  | **Mean±SD** | **Mean±SD** |  |
| **Trabecular Bone Properties** | **Femur Length (mm)** | ns | **** | ns | 14.44±0.21 | 14.27±0.15 | ns | 15.59±0.16 | 15.43±0.10 | ns | 15.03±0.32 | 15.07±0.11 | ns |
|  | **BV/TV (%)** | *** | **** | **** | 31.01±3.02 | 17.51±2.7 | **** | 24.10±2.38 | 16.16±3.02 | ** | 7.68±2.67 | 6.72±4.32 | ns |
|  | **Conn.D. (1/mm^3^)** | ** | **** | ** | 242.6±15.2 | 199.6±23.2 | **** | 115.6±4.3 | 120.7±15.2 | ns | 24.7±5.1 | 13.8±6.8 | ns |
|  | **SMI** | *** | **** | **** | 1.21±0.26 | 2.26±0.23 | **** | 1.26±0.18 | 2.20±0.20 | **** | 2.96±-.34 | 3.12±0.35 | ns |
|  | **Tb.N (1/mm)** | *** | **** | **** | 6.39±0.20 | 5.31±0.22 | **** | 4.76±0.09 | 4.54±0.09 | ns | 2.98±-.18 | 2.52±0.34 | ** |
|  | **Tb.Th (mm)** | ns (0.0565) | ns | ns (0.0553) | 0.066±0.005 | 0.050±0.033 | * | 0.066±0.004 | 0.056±0.006 | ns | 0.063±0.012 | 0.068±0.021 | ns |
|  | **Tb.Sp (mm)** | ** | **** | **** | 0.146±0.005 | 0.181±0.008 | * | 0.196±0.005 | 0.209±0.006 | ns | 0.324±0.022 | 0.399±0.051 | **** |
| **Cortical Bone Properties** | **Ct.Th (mm)** | *** | **** | ** | 0.164±0.006 | 0.136±0.01 | **** | 0.175±0.008 | 0.182±0.012 | ns | 0.178±0.008 | 0.169±0.007 | ns |
|  | **Ct.TMD (mgHA/cm^3^)** | ns | **** | ns | 1049±8 | 1038±3 | ns | 1109±14 | 1118±7 | ns | 1160±18 | 1159±20 | ns |
|  | **Ct.Ar (mm^2^)** | ** | **** | **** | 0.842±0.044 | 0.611±0.062 | **** | 0.949±0.04 | 0.822±0.060 | ** | 0.885±0.028 | 0.812±0.054 | ns |
|  | **Ma.Ar (mm^2^)** | ** | ns | **** | 1.395±0.077 | 1.155±0.051 | *** | 1.484±0.116 | 1.030±0.037 | **** | 1.365±0.207 | 1.273±0.123 | ns |
|  | **Tt.Ar (mm^2^)** | ** | * | **** | 2.237±0.108 | 1.766±0.104 | **** | 2.433±0.104 | 1.853±0.082 | **** | 2.250±0.219 | 2.084±0.165 | ns |
|  | **Ct.Ar/Tt.Ar (%)** | **** | **** | ns | 37.65±1.17 | 34.52±1.83 | * | 39.08±2.70 | 44.36±1.60 | ** | 39.58±3.10 | 38.99±1.66 | ns |
|  | **pMOI (mm^4^)** | ** | **** | **** | 0.512±0.047 | 0.303±0.047 | **** | 0.633±0.040 | 0.404±0.041 | **** | 0.539±0.064 | 0.460±0.057 | * |
|  | **Imax (mm^4^)** | *** | **** | **** | 0.341±0.033 | 0.205±0.034 | **** | 0.435±0.027 | 0.276±0.025 | **** | 0.364±0.033 | 0.308±0.028 | * |
|  | **Imin (mm^4^)** | * | ** | **** | 0.171±0.018 | 0.098±0.015 | **** | 0.197±0.014 | 0.128±0.016 | *** | 0.175±0.032 | 0.152±0.031 | ns |

**Figure 4 – Source Data 3.** Full quantitative parameters for histomorphometry corresponding to representative selection in Figure 4c. Descriptive statistics reported as mean ± standard deviation.

| **Histomorphometry**  **Bone Parameters**  **in Tibia** | **2 Months** | | | **13 Months** | | |
| --- | --- | --- | --- | --- | --- | --- |
|  | **WT** | **TG** | ***P* Value** | **WT** | **TG** | ***P* Value** |
|  | **Mean±SD** | **Mean±SD** |  | **Mean±SD** | **Mean±SD** |  |
| **BV/TV (%)** | 14.6±2.61 | 9.52±2.06 | 0.0007*** | 5.04+1.30 | 2.96+2.29 | 0.0821 |
| **Tb.Th (µm)** | 37.1±4.97 | 31.1+4.21 | 0.0209* | 38.3+6.67 | 32.7+8.43 | 0.2332 |
| **Tb.N (/mm)** | 3.93±0.52 | 3.05+0.47 | 0.0032** | 1.31+0.24 | 0.85+0.55 | 0.0908 |
| **Tb.Sp (µm)** | 223±40.0 | 305+49.0 | 0.0026** | 774+149 | 1801+1403 | 0.1047 |
| **MAR (µm/day)** | 2.25±0.25 | 1.87+0.22 | 0.0137* | 0.67+0.04 | 0.69+0.35 | 0.8926 |
| **MS/BS (%)** | 51.9±3.15 | 48.0+3.44 | 0.0574 | 25.1+12.0 | 22.4+5.72 | 0.6608 |
| **BFR/BV (%/day)** | 6.35±0.68 | 6.34+1.13 | 0.9912 | 0.96+0.40 | 1.04+0.67 | 0.7920 |
| **BFR/BS (µm^3^/µm^2^/day)** | 1.17±0.15 | 0.90+0.12 | 0.0046** | 0.17+0.08 | 0.17+0.12 | 0.9525 |
| **Ob.S/B.Pm (%)** | 22.2±3.67 | 18.1+3.45 | 0.0367* | 4.71+1.59 | 5.91+3.78 | 0.4892 |
| **N.Ob./B.Pm (/mm)** | 17.7±3.07 | 14.6+2.82 | 0.0543 | 4.32+1.25 | 5.48+3.23 | 0.4321 |
| **OS/BS (%)** | 16.3±4.82 | 12.9+2.12 | 0.0862 | 4.42+4.27 | 7.12+6.80 | 0.4288 |
| **O.Th (µm)** | 3.22±0.30 | 2.83+0.21 | 0.0106* | 2.63+0.37 | 2.32+0.65 | 0.3402 |
| **Oc.S/B.Pm (%)** | 18.0±1.92 | 18.2+3.18 | 0.8315 | 10.8+3.24 | 11.2+3.34 | 0.8500 |
| **N.Oc/B.Pm (/mm)** | 6.60±0.71 | 6.72+1.29 | 0.82 | 4.06+1.06 | 5.17+1.55 | 0.1817 |
| **ES/BS (%)** | 4.21±1.39 | 3.48+1.25 | 0.2873 | 1.20+0.74 | 0.83+0.92 | 0.4561 |
