# Supplementary material for: Irisin directly stimulates osteoclastogenesis and bone resorption in vitro and in vivo
Source: eLife. 2020 Aug 11;9:e58172. doi: 10.7554/eLife.58172 (PMC7444909; doi:10.7554/eLife.58172)
Supplement: Transparent reporting form [file elife-58172-transrepform.pdf]

eLIFE
1st Floor
24 Hills Road
Cambridge CB2 1JP, UK
P 01223 855340
W elifesciences.org
T @elife

Transcriptional co-activator PGC-1 alpha drives the formation of slow-twitch muscle fibres.

eLife's transparent reporting form

We encourage authors to provide detailed information *within their submission* to facilitate the interpretation and replication of experiments. Authors can upload supporting documentation to indicate the use of appropriate reporting guidelines for health-related research (see EQUATOR Network), life science research (see the BioSharing Information Resource), or the ARRIVE guidelines for reporting work involving animal research. Where applicable, authors should refer to any relevant reporting standards documents in this form.

If you have any questions, please consult our Journal Policies and/or contact us: editorial@elifesciences.org.

Sample-size estimation

- You should state whether an appropriate sample size was computed when the study was being designed
- You should state the statistical method of sample size computation and any required assumptions
- If no explicit power analysis was used, you should describe how you decided what sample (replicate) size (number) to use

Please outline where this information can be found within the submission (e.g., sections or figure legends), or explain why this information doesn't apply to your submission:

The estimation of sample size via *a priori* power analyses based on preliminary data is described at the end of the Methods section under the heading *Experimental Design and Data Analysis*, with explanation of deviation from estimated numbers due to experimental factor limitations detailed where appropriate.

Replicates

- You should report how often each experiment was performed
- You should include a definition of biological versus technical replication
- The data obtained should be provided and sufficient information should be provided to indicate the number of independent biological and/or technical replicates
- If you encountered any outliers, you should describe how these were handled
- Criteria for exclusion/inclusion of data should be clearly stated
- High-throughput sequence data should be uploaded before submission, with a private link for reviewers provided (these are available from both GEO and ArrayExpress)

Please outline where this information can be found within the submission (e.g., sections or figure legends), or explain why this information doesn't apply to your submission:

The definition and number of biological vs. technical replicates, and details of experimental repeats and the handling of outliers are described at the end of the Methods section under the heading *Experimental Design and Data Analysis*. Individual sample data is represented in each figure with column and scatterplot overlay graphs, with samples sizes for each experiment reported in the figure legends.

eLife Sciences Publications, Ltd is a limited liability non-profit non-stock corporation incorporated in the State of Delaware, USA, with company number 5030732, and is registered in the UK with company number FC030576 and branch number BR015634 at the address 1st Floor, 24 Hills Road, Cambridge CB2 1JP | August 2014

1st Floor
24 Hills Road
Cambridge CB2 1JP, UK

P 01223 855340
W elifesciences.org
T @elife

eLife Sciences Publications, Ltd is a limited liability non-profit non-stock corporation incorporated in the State of Delaware, USA, with company number 5030732, and is registered in the UK with company number FC030576 and branch number BR015634 at the address 1st Floor, 24 Hills Road, Cambridge CB2 1JP | August 2014

Statistical reporting

- Statistical analysis methods should be described and justified
- Raw data should be presented in figures whenever informative to do so (typically when N per group is less than 10)
- For each experiment, you should identify the statistical tests used, exact values of N, definitions of center, methods of multiple test correction, and dispersion and precision measures (e.g., mean, median, SD, SEM, confidence intervals; and, for the major substantive results, a measure of effect size (e.g., Pearson's r, Cohen's d)
- Report exact p-values wherever possible alongside the summary statistics and 95% confidence intervals. These should be reported for all key questions and not only when the p-value is less than 0.05.

Please outline where this information can be found within the submission (e.g., sections or figure legends), or explain why this information doesn't apply to your submission:

The selection of methods for statistical analysis are described and justified at the end of the Methods section under the heading *Experimental Design and Data Analysis*. As the data sets involved are relatively small, all raw data are presented directly in figure graphs as a scatterplot overlay on the summary column graphics for mean and standard deviation. The statistical tests used for each experiment are broken down in the Methods section, with specific N values reported there and in the figure legends. "Less-than" P values are provided for succinctness in the figure legends, with exact p-values provided in the results section for all relevant comparisons. Summary statistics are provided in the supplemental tables to each figure.

(For large datasets, or papers with a very large number of statistical tests, you may upload a single table file with tests, Ns, etc., with reference to sections in the manuscript.)

Group allocation

- Indicate how samples were allocated into experimental groups (in the case of clinical studies, please specify allocation to treatment method); if randomization was used, please also state if restricted randomization was applied
- Indicate if masking was used during group allocation, data collection and/or data analysis

Please outline where this information can be found within the submission (e.g., sections or figure legends), or explain why this information doesn't apply to your submission:

The nature of experiments in this study, with exogenous treatment of in vitro cultures or comparison of transgenic versus wild type mice, did not necessitate group allocation methods beyond direct assignment by the investigator during treatment in the former case. Masking was employed to confirm quantifications for the experiments that were based on investigator interpretation of osteoclast identification, as described in the Methods section under *Osteoclast Counts.*

Additional data files ("source data")

- We encourage you to upload relevant additional data files, such as numerical data that are represented as a graph in a figure, or as a summary table
- Where provided, these should be in the most useful format, and they can be uploaded as "Source data" files linked to a main figure or table
- Include model definition files including the full list of parameters used
- Include code used for data analysis (e.g., R, MatLab)

eLife Sciences Publications, Ltd is a limited liability non-profit non-stock corporation incorporated in the State of Delaware, USA, with company number 5030732, and is registered in the UK with company number FC030576 and branch number BR015634 at the address 1st Floor, 24 Hills Road, Cambridge CB2 1JP | August 2014

- Avoid stating that data files are “available upon request”

Please indicate the figures or tables for which source data files have been provided:

As the data sets generated in these experiments were small in scope, the individual source data have been represented within figure graphs themselves as scatterplot overlays, with the summary data provided in supplement tables. No novel or custom models or source code were employed.
